# Field survey insights and performance assessment of water-in-glass evacuated tube solar water heaters in Burkina Faso

**DOI:** 10.1038/s41598-026-48447-w

**Published:** 2026-04-11

**Authors:** Kokouvi Edem N’Tsoukpoe, Claude Sara Lekombo, Malicki Zorom, Komlan Gagno José N’Tsoukpoe, Frédéric Kuznik

**Affiliations:** 1 Laboratory for Renewable Energy and Energy Efficiency (LabEREE), Department of Electrical, Industrial and Energy Engineering, International Institute for Water and Environmental Engineering (2iE), 01 BP 594, Ouagadougou 01, Burkina Faso; 2 Laboratory of Water, Hydro-Systems and Agriculture (LEHSA), Department of Engineering Science and Technology, International Institute for Water and Environmental Engineering (2iE), 01 BP 594, Ouagadougou 01, Burkina Faso; 3 CETHIL UMR5008, INSA Lyon, University of Lyon, 69622 Villeurbanne, France

**Keywords:** Solar water heater, Water-in-glass evacuated tube collector, Float valve failure, User satisfaction, Technology assessment, Burkina Faso, Energy science and technology, Engineering

## Abstract

Water-in-glass evacuated tube collectors (WiG-ETCs) now dominate Ouagadougou’s solar water-heating market as locally manufactured flat plate collectors (FPCs) continue to decline. This study evaluates the relevance and long-term viability of WiG-ETCs in Burkina Faso by assessing their potential, challenges, and prospects for sustained adoption. A field survey of 31 operational systems in Ouagadougou—24 households and one seminary—was conducted in April 2024, supported by a literature review and market analysis. The inspection covered system functionality, maintenance history, tank material, tilt angle, and visible signs of deterioration. Results show that 94% of systems were operational at the time of the survey. The most frequent issues were malfunctioning float valves (35%) and water leakages (29%). Stainless-steel tanks demonstrated markedly higher durability, with several systems operating for more than 14 years. User satisfaction was high: 97% of respondents reported being satisfied with their investment, and 74% reported year-round hot-water availability. Older or rust-affected systems exhibited greater maintenance needs. Overall, WiG-ETCs demonstrate high operational reliability and strong user satisfaction in Burkina Faso. To secure this potential, key priorities include addressing prevalent float-valve failures and promoting corrosion-resistant stainless-steel tanks to enhance system longevity.

**Supplementary Information:**

The online version contains supplementary material available at 10.1038/s41598-026-48447-w.

## Introduction

Identifying the appropriate technology for harnessing renewable energy is one of the most crucial considerations for energy decision-makers. In the field of renewable energy, technology choices and promotion strategies must be made carefully to avoid negative perceptions that could hinder both adoption and policy development^1–3^. Solar water heaters (SWHs) are among the most important yet low-cost technologies for reducing dependence on fossil fuels and decreasing greenhouse gas emissions. Their relevance is particularly high in Burkina Faso, where over 80% of the energy demand is met by traditional biomass^4^, contributing to deforestation and health challenges. In Burkina Faso, situated at the heart of the Sahel, the local SWH manufacturing industry, which has been traditionally based on flat plate collectors (FPCs), has experienced a significant decline compared to the growing importation of evacuated tube collectors (ETCs), notably water-in-glass (WiG) ETCs, over the past decade^5^. A study^6^ conducted in 2010 in Ouagadougou offers an historical context on the early deployment of SWHs. At that time, most systems were locally manufactured FPCs, and users reported failures as well as difficulties in obtaining reliable maintenance support. These challenges contributed to the discontinuation of several installations and shaped the initial perception of SWH technologies in the country. Despite abundant solar resources, the adoption level of SWHs in Burkina Faso remains very low^5,7^, with usage concentrated mainly in larger cities^8,9^. In rural and peri-urban areas—home to about 70% of the population—awareness and demand for SWH technology remain almost non-existent^10^.

WiG-ETCs have become the dominant solar water heating technology in Burkina Faso. Their growing popularity is driven mainly by their affordability and by the import tax exemption on renewable energy equipment introduced in 2013^5^. WiG-ETCs are typically half the cost of equivalent FPC systems (Table 1) (in this study, all financial values are presented in Euros (€) to ensure international clarity and consistency. The local currency in Burkina Faso is the West African CFA Franc (XOF), which is pegged to the Euro at a fixed exchange rate of 1 € = 655.957 XOF. This fixed rate has been consistently used throughout our analysis), making them more accessible to a broader population. A recent study by the authors, published in 2025, documented the technical and economic performance of selected thermosiphon SWHs in Ouagadougou and Lomé, including WiG-ETCs^11^. Long-term monitoring results showed that WiG-ETCs had the shortest payback periods (2.4–2.9 years when replacing electric water heaters)^11^. Despite not being certified, the WiG-ETCs outperformed certified systems in real-world efficiency, which is strongly influenced by factors such as installation quality, system design, and maintenance^11^. The study established the technical potential but simultaneously highlighted a critical gap. The broader landscape of WiG-ETC uptake, long-term reliability, user satisfaction, and recurrent failures at the household level remains largely unexplored. The long-term success of SWH programmes depends not only on technical performance but also on user acceptance, routine maintenance, and alignment with local expectations—factors frequently overlooked in the literature^1^.

**Table 1:** Average cost of thermosiphon solar water heaters and electrical heaters in Ouagadougou. For imported systems, these are displayed prices, all taxes included, without any reduction or installation costs, and have been obtained from the six main SWH distributors (importers) in Ouagadougou on May 28th, 2024.

System	Capacity [L]	Collector aperture area [m^2^] ^a^	Aperture to capacity ratio [m^2^/m^3^]	Cost [€]	Cost per capacity [€/L]
SWH FPC, pressure, indirect, locally manufactured	100	1.8	18.0	686–838	6.86–8.38
	200	3.6	18.0	1220	6.10
SWH FPC, stainless steel, pressure, (indirect), Solar Keymark, imported	150	1.88	12.5	909	6.06
	180	2.38	13.2	1 067	5.93
	280	3.76	13.4	1 600	5.71
SWH FPC (indirect), imported	150	-	-	1 067	7.11
	200	-	-	1 509	7.55
	300	-	-	1 753	5.84
SWH WiG-ETC stainless steel, imported	100	1.0	10.3	421	4.21
	120	1.2	10.3	456	3.80
	150	1.5	10.3	531	3.54
	180	1.9	10.3	573	3.18
	200	2.1	10.3	579	2.90
	240	2.5	10.3	747	3.11
	300	3.1	10.3	835	2.78
	360	3.7	10.3	867	2.41
SWH WiG-ETC steel, imported	100	1.0	10.3	267–453	2.67–4.53
	150	1.5	10.3	381–605	2.54–4.03
SWH WiG-ETC stainless steel, pressure, indirect (DHW inside heat exchanger), + magnesium rod, imported	150	1.5	10.3	534	3.56
	200	2.1	10.3	579	2.90
Tank-based electrical water heaters (accumulators), imported	30	-	-	175–208	5.83–6.93
	50	-	-	191–242	3.82–4.84
	80	-	-	252–267	3.15–3.34
	100	-	-	267	2.67

^a^ The aperture area has been adopted because it is the most crucial area when considering energy conversion efficiency.

Globally, research on ETCs is extensive but primarily focused on laboratory evaluations, thermal performance optimisation, and advanced materials such as nanofluids and phase change materials (PCMs)^12^. Field-based studies addressing maintenance issues, life expectancy, real-world degradation, or socio-economic impacts remain limited. This lack of empirical data is even more pronounced in West Africa, where most SWHs are installed without performance indicators, making it difficult for engineers, policymakers, and consumers to make informed decisions. Critically, studies from other contexts highlight that managing user expectations with accurate information is essential for long-term technology acceptance^1^. Fundamental information such as life expectancy, potential recurrent failures and corresponding frequency, or real operational constraints is generally unavailable, and little is known about their adaptation to the local context. Given this context, there is a need to analyse the operational status, maintenance challenges, and user satisfaction associated with WiG-ETCs for domestic hot water (DHW) in a Sahelian context such as Burkina Faso. Such insights are crucial for supporting policy development, guiding consumer choices, informing economic analysis, and designing realistic incentive schemes or quality-assurance frameworks. Understanding how WiG-ETCs perform under real use conditions—and whether they meet user expectations—is therefore essential for scaling up sustainable water heating solutions.

Against this background, this study aims to explore the potential of WiG-ETC SWHs in Burkina Faso, by focusing on their feasibility, challenges, and user satisfaction. The specific objectives are to (i) assess the state of WiG-ETC technologies in Burkina Faso; (ii) identify the main challenges and failures exhibited by these systems; (iii) evaluate user satisfaction; and (iv) provide recommendations to support the development and sustainability of the solar water heating sector in Burkina Faso and in regions with similar characteristics.

The rest of the paper is organised as follows. “Lessons learned from the literature on water-in-glass evacuated tube collectors” provides background information and a detailed literature review concerning WiG-ETCs. An overview of solar water heating technology development and applications in Burkina Faso is provided in “Overview of solar water heating technology development and applications in Burkina Faso”. “Survey of water-in-glass evacuated tube solar water heaters” firstly describes the methodology employed in the survey conducted on approximately 30 households in Ouagadougou. Then, it presents and discusses the findings and outlines the study’s limitations and areas for further research. “Conclusion, practical implications and outlook” concludes the paper by summarising the key insights and recommendations.

## Lessons learned from the literature on water-in-glass evacuated tube collectors

### Introduction to water-in-glass evacuated tube collectors

**Fig. 1:**
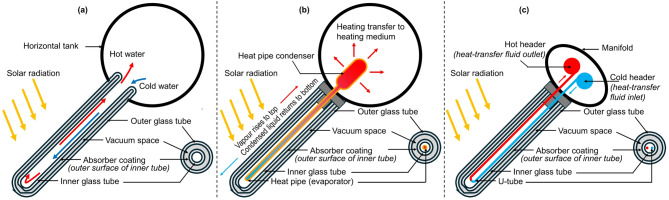
Schematic representation and cross-section views of the main types of evacuated tube collectors: (**a**) water-in-glass (WiG), (**b**) U-pipe, and (**c**) heat-pipe collectors.

There are three main types of ETC: WiG, U-type, and heat pipe^12–15^(Fig. 1). WiG-ETCs operate as thermosiphon systems (Fig. 1a): heated water circulating directly within the evacuated glass tubes rises to the storage tank, while cooler water from the tank flows downward for heating in a continuous cycle^13–15^. In WiG-ETCs, heat is absorbed through the walls of the glass tubes^14,15^. In heat pipe ETCs, heat is transferred to the heating fluid by a heat transfer fluid—typically an alcohol, that undergoes an evaporation-condensation cycle within the heat pipe—through the condenser section (Fig. 1b)^13,15^. In U-type ETCs (Fig. 1c), heat is absorbed as the heating fluid flows through U-shaped copper tubes^15^.

WiG-ETCs, also known as wet ETCs, offer a unique advantage due to their all-glass construction. This design eliminates the need for an additional glass layer for heat transfer, thereby reducing potential leakage issues. Consequently, WiG-ETCs tend to be more cost-effective than other types, such as heat pipes and U-type ETCs^13,16,17^.

**Fig. 2:**
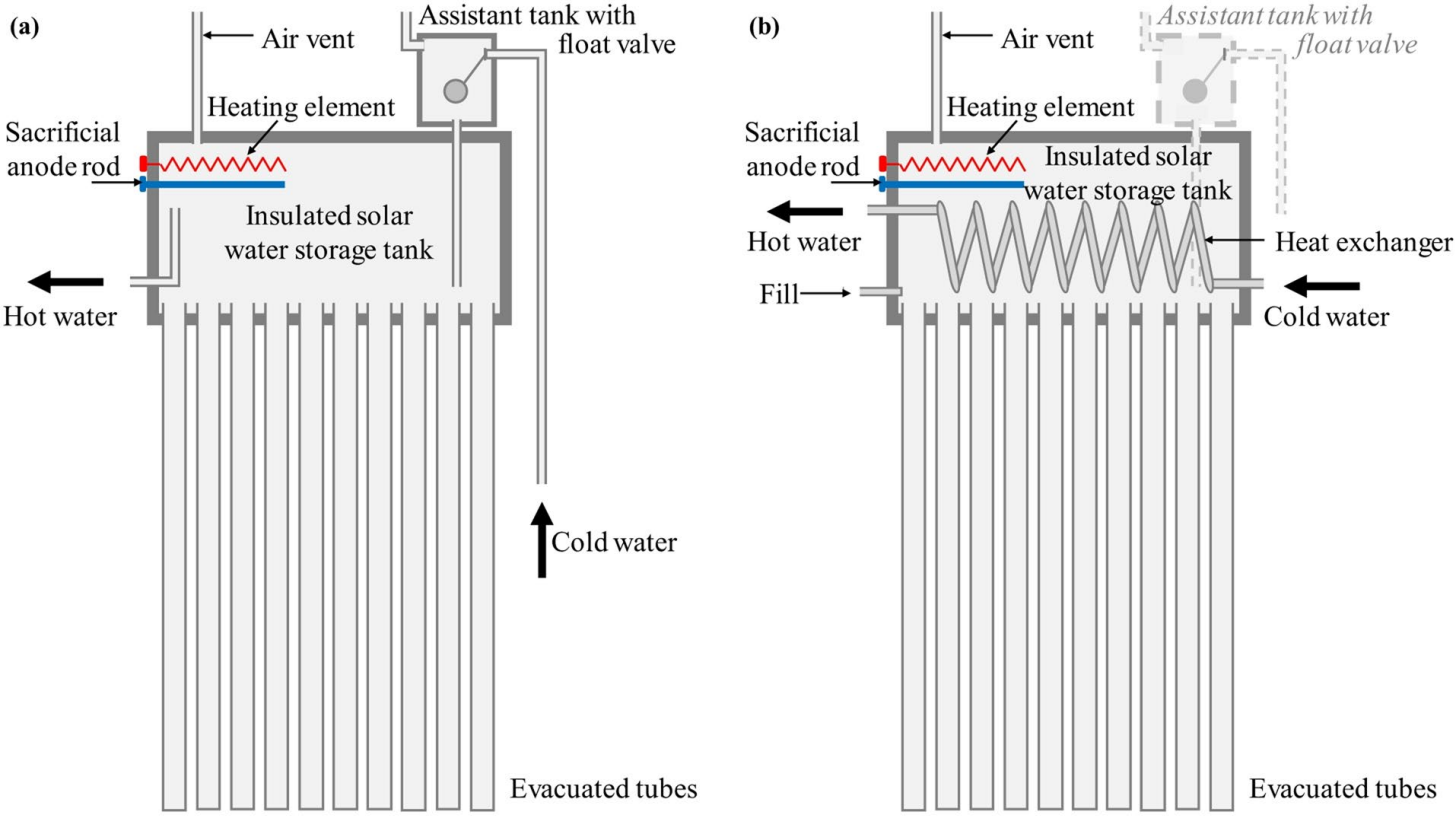
(**a**) Direct and (**b**) indirect WiG-ETC SWH. The heating element and the anode rod are optional. In the indirect system, an assistant tank is not needed.

There are two types of WiG-ETC SWHs, namely, direct and indirect systems (Table 2). In direct systems (Fig. 2a), the water in the storage tank is used directly by the user. Since the tank operates at atmospheric pressure, the service pressure is limited to prevent glass breakage. Typically, an assistant tank with a float valve is mounted on the storage tank to automatically refill it with cold water when hot water is drawn. In indirect WiG-ETCs, a heat exchanger within the storage tank separates the solar loop (water circulating within the glass tubes and kept in the tank) from the DHW circulating through the heat exchanger (Fig. 2b). This setup allows indirect systems to handle high pressure on the user side while maintaining atmospheric pressure on the solar side. This offers the possibility of benefiting from water supply network pressure at the tap point, avoiding water stagnation-related issues (e.g., legionella and other bacteria growth) and poor water quality in the tank (e.g., scaling, corrosion). Since the user hot water circulates inside the coil of the heat exchanger and exchanges heat instantaneously with the water in the tank, the size of the heat exchanger needs to be sufficiently large to allow efficient heat exchange when hot water is needed. Hence, indirect systems tend to be more expensive than direct systems.

**Table 2:** Comparison of direct and indirect WiG-ETCs.

Parameter	Direct WiG-ETC	Indirect WiG-ETC
Inherent system operating pressure	Low-pressure, gravity-fed system. (near-atmospheric) to avoid glass-tube damage); cannot operate in a pressurised DHW network	Pressurised system. Operates at mains DHW pressure i.e. can utilise the pressure of the mains water network
Service pressure & ability to supply service points	The available pressure at the tap is solely determined by the vertical height (water head) between the tank outlet and the service point: service pressure limited to gravity head; can supply only service points located below the SWH	Service pressure follows mains pressure; can supply service points above the SWH as long as adequate network pressure is available
Performance during low network pressure	Good – hot water remains accessible from the storage tank	Poor – insufficient pressure prevents effective flow and heat exchange
Performance during complete water shortages	Good – stored DHW is available for use	Very poor – system is inoperable without water supply. Auxiliary pumps are useless
Performance during power outages	Good	If a pressurising device is used, then DHW shortage during power outages, which are quite frequent^18,19^
Hot water storage & Pressure resilience	Stores DHW directly in tank; provides high resilience during low pressure or shortages	No direct DHW storage; requires adequate mains pressure (typically ~ 1.5–3 bar) to circulate water through heat exchanger
Water quality & Contamination risk	Higher risk – DHW stagnates in tank; periodic cleaning is recommended. Legionella risk exists but was not detected locally^20^	Lower risk – closed solar loop; fresh DHW is heated instantaneously via heat exchanger, reducing stagnation
Maintenance focus	Tank cleaning, anode rod, float valve – components exposed to raw water	Heat exchanger integrity, pump, valves – maintenance of the pressurised solar loop and auxiliary components
Typical initial cost (Table 1)	Lower – simpler design with fewer components	Higher – additional cost of heat exchanger, and sometime a pump

**Fig. 3:**
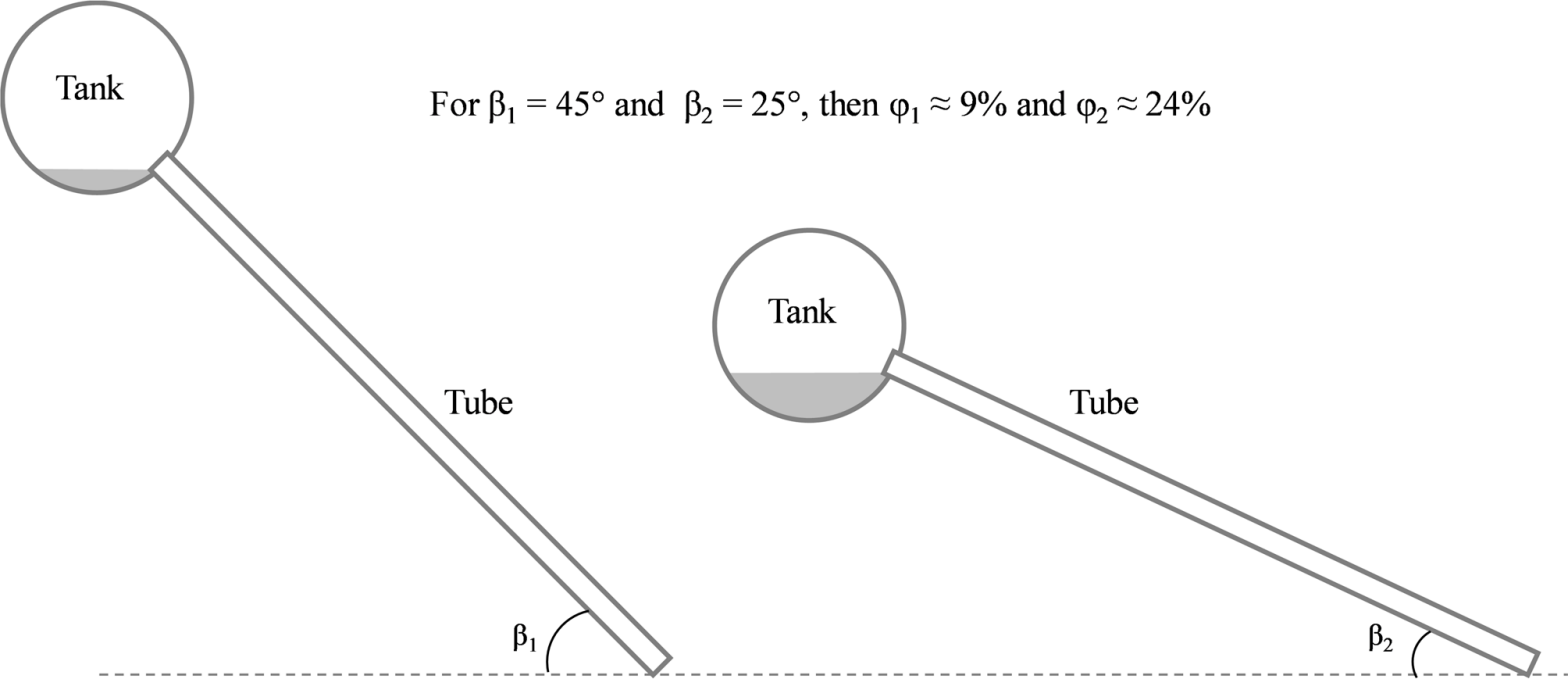
Effect of the collector tilt angle on the volume of water below the insertion point, considering a fixed storage tank diameter.

The tubes in the WiG-ETCs are inserted into the tank at an angle along the diameter plane, which prevents the thermosyphon circulation from reaching the lower section of the tank. With a 45° tube inclination, around 9% of the tank volume is below the insertion point and is not directly heated^21^(Fig. 3). As the tube inclination decreases, the portion of the tank volume not directly heated by the water circulating within the evacuated tubes significantly increases^21,22^. Neglecting the effect of the internal diameter of the tube, the fraction φ of the tank volume below the point where the tubes are inserted can be calculated at a tilt angle β [°] (1):

1 $$\:\phi\:=\frac{1}{2}-\frac{\beta\:}{180}-\frac{{sin}\left(2\beta\:\right)}{2\pi\:}$$

### Why are WiG-ETCs so attractive?

The attractiveness of WiG-ETCs in the global market is driven by a combination of strong thermal performance, durability, and economic factors. They are increasingly popular for solar water heating due to their high thermal efficiency, especially at high temperatures, making them suitable for solar cooling, desalination, and industrial processes^13,23^. This performance is often superior to FPCs under various conditions. For instance, they exhibit greater efficiency than FPCs at low incidence angles, which typically gives them an advantage in terms of all-day performance^13,23^. Statistical analysis of 909 solar thermal collectors data available in the Solar Keymark Database as of November 2024^24^ confirms that, in general, flat-plate collectors (FPCs) exhibit higher optical efficiency parameters, but their thermal efficiency declines more steeply with operating temperature than that of evacuated tube collectors (ETCs). This makes ETCs more effective in windy or cold conditions, as already reported by Zambolin and Del Col^25^. Each tube directly heats water, with a vacuum between glass layers minimising heat loss and ensuring performance even on cloudy days^23^. This vacuum also protects the absorber plate from environmental conditions, extending its lifespan.

Beyond performance, practical and economic advantages are significant. The borosilicate glass tubes resist corrosion and allow wind to pass through, reducing structural stress and the need for robust mounts, which is ideal for the Sahel’s windy regions. Their modular design facilitates transport, handling, and installation, particularly in remote areas^23^. Furthermore, their simple design leads to minimal maintenance requirements and high reparability, as Mangal et al.^17^ note that a single broken tube can be replaced cheaply without changing the entire collector. The widespread production, especially in China, has drastically lowered costs, making them more accessible and affordable^16,23^, a key reason for their dominance in price-sensitive markets. This economic advantage is clear from various analysis, including comparative studies. For instance, an analysis in China suggests that the initial cost of a WiG-ETC system may be five times less expensive to a comparable FPC system, leading to a much shorter payback period^23^. Chow et al.^26^ compared the performance of a WiG-ETC and to a similar heat pipe ETC in eight cities, representing the seven climate regions of China. The WiG-ETCs exhibited shorter (12‒20%) cost payback period in all these cities, especially in the warmer regions, although its thermal efficiency is lower (5‒13%) under the same weather condition. So, other ETC types like heat pipes and U-pipe collectors can offer higher thermal performance, as Gao et al.^27^ demonstrated that U-pipe ETCs achieve 25–35% higher energy storage, representing a clear performance-cost trade-off. In the Lebanese context, Hayek et al.^28^ drew similar conclusion while highlighting that heat pipe ETCs can be 15‒20% more efficient than WiG-ETCs.

### What are the common challenges and failures of ETCs?

**Fig. 4:**
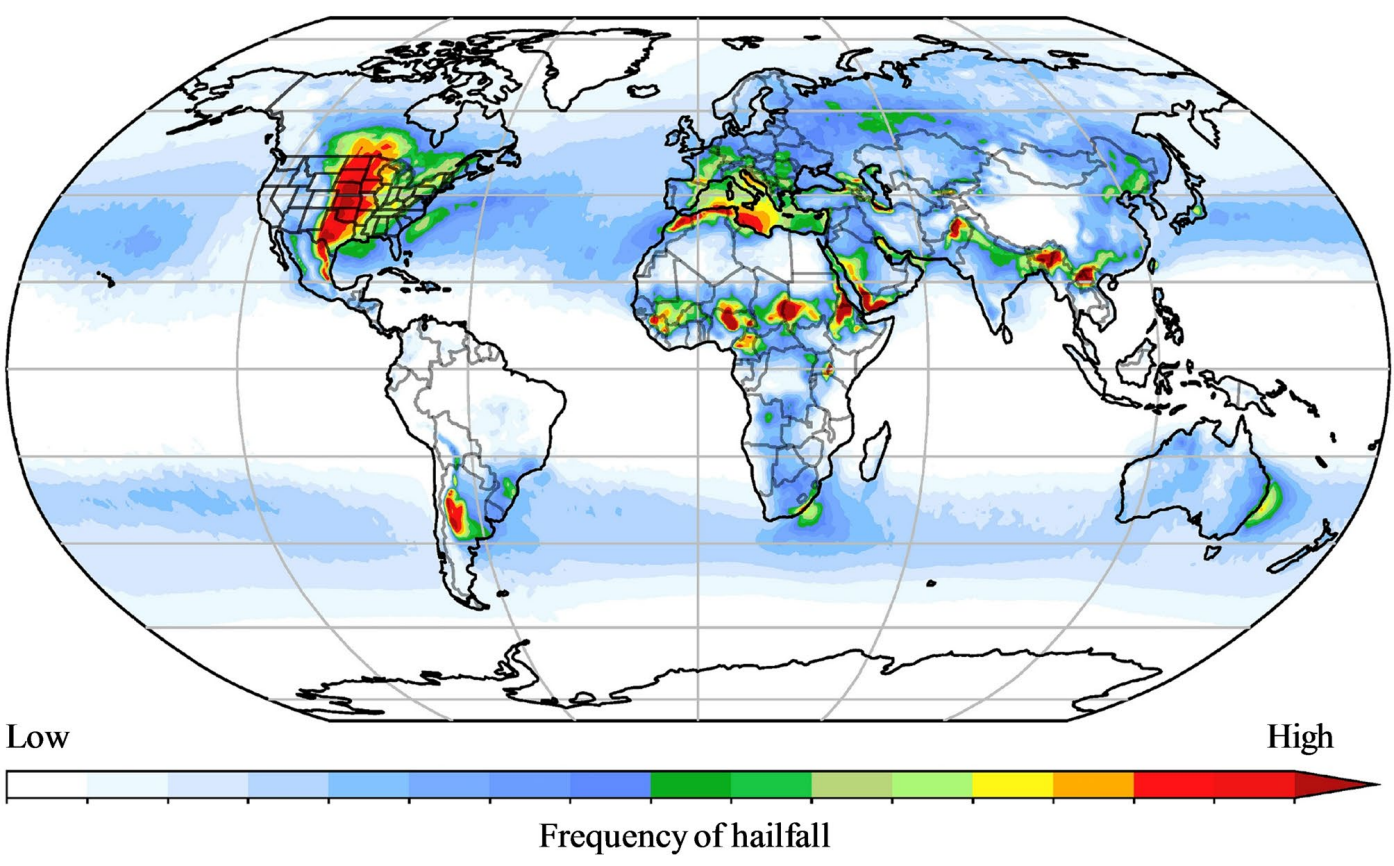
Risk areas for hail, based on the atmospheric conditions in which large hail (> 2.5 cm in diameter) generally forms^30^.

Despite their advantages, the literature also highlights several inherent challenges and common failure modes of WiG-ETC technology. One major issue is the fact that the collector tubes are very fragile and easily damaged, making them unsuitable for hail-prone areas^23^. In sub-Saharan Africa, hail is primarily linked to thunderstorms in the Intertropical Convergence Zone (ITCZ), i.e., West Africa and East Africa (Uganda, Kenya, Tanzania) but also to regions in South Africa (Fig. 4). For instance, Netshiozwi^29^ reported that the majority of the SWHs installed in the City of Tshwane through the South African Solar Water Heating Programme were damaged during hailstorms and were not repaired, impacting sustainability, as the systems operated for only a brief period.

The fragility of the pipe also limits their operation to low-pressure systems, as their design cannot withstand high pressures in the tank without risking complete fluid loss if a single tube breaks. Particularly in direct WiG-ETCs (Fig. 2a), the water in the tank serves both the solar loop and DHW needs without a separate heat exchanger. This setup operates at atmospheric pressure, which limits the pressure delivered at the tap point.

Other operational challenges often relate to water quality and component durability. The corrosion of components and heat transfer inefficiencies due to potential scaling, especially with hard water, have been reported by Kumbhar^31^. Tank leakages are common and are often caused by sealing gasket or washer failures, leading to corrosion, algae formation, and scaling^31^. Another concern is the accumulation of salts inside the collector tubes, which can obstruct water flow and cause glass tubes to burst due to superheated trapped water forming high-pressure steam^31^.

### Installation tilt angle and other design considerations

The tilt angle of SWHs significantly affects their performance. Regarding WiG-ETCs, experimental studies have shown that the tilt angle of WiG-ETCs has a negligible impact on the water flow through the tubes^22,32,33^. While the collector tilt angle influences daily collectible radiation and solar heat gain, it has minimal impact on heat extraction from evacuated tubes to the storage tank and daily thermal efficiency^22^. For instance, Tang et al.^22^ experimentally found while a WiG-ETC SWH with a 22° tilt maintained a continuous water circulation loop, a system at 46° experienced mixing between cold and hot water, which reduced thermosiphon efficiency. This mixing reduced the efficiency of thermosiphonic circulation, indicating that increasing the tilt angle does not positively affect heat removal or water circulation within the tubes. The study concluded that to maximise annual heat gain, the collector should be inclined to optimise annual solar radiation collection. Budihardjo and Morrison^34^ reinforced this, finding that the annual solar fraction increased by only 1.5% when the tilt angle was increased from 22° to 45° in Sydney (latitude = -34°), although the savings during winter were 12% greater at a 45° inclination. After testing more than 900 WiG-ETC SWHs at the National Center for Quality Supervision and Testing of Solar Heating Systems (Beijing, China), Zhang et al.^33^ confirm the previous studies stating that the tilt angle does not significantly affect the performance of the WiG-ETC SWHs.

From their testing of more than 900 WiG-ETC SWHs, Zhang et al.^33^ found that the optimum ratio of tank volume to collector area for solar water heater is 57 to 72 L/m^2^. Several studies have been proposed to improve the energy performance of WiG-ETC, including the use of PCM or the incorporation of nanofluids^12,14^.

##  Overview of solar water heating technology development and applications in Burkina Faso

### Energy context in Burkina Faso

Energy poverty in Burkina Faso remains high, with the country heavily reliant on traditional biomass, which accounts for 73% of total energy supply^35^. This dependence contributes to widespread deforestation and exacerbates the impacts of climate change in a region already classified as highly vulnerable. Burkina Faso ranks among the ten countries with the lowest Human Development Index worldwide and has an electrification rate of only 27%^4^, with frequent load shedding, particularly during the hot season. Since the late 2010s, the country has also faced significant security challenges and political instability, which complicate development efforts, further constraining energy access^36^.

Modern energy production in Burkina Faso is limited. Electricity represents less than 4% of final energy consumption^35,37^, and national generation relies largely on imported fossil fuels (78%), primarily diesel and heavy fuel oil^35,38^. Hydropower contributes a modest 8%, while solar PV has been growing rapidly, accounting for 14% of electricity generation^38^. To satisfy its domestic electricity needs, Burkina Faso relies on imports from neighbouring countries, which account for 58% of its total electricity consumption in 2023^38^. Since 2017, several solar power plants have been commissioned, increasing installed solar capacity from less than 40 MW in 2015 to over 220 MW by early 2025, positioning solar as the country’s main indigenous source of modern energy. Despite recent ambitions—signature of a roadmap for the development of Russian-Burkinabe cooperation concluded on in March 2024^39,40^—, Burkina Faso has no nuclear energy production and continues to rely heavily on electricity imports from neighbouring countries, which supply 58% of the national electricity mix^38^. These trends highlight the structural vulnerability of the national energy system and underscore the strategic importance of solar technologies—thermal and photovoltaic—as the only scalable domestic energy resource.

The country benefits from abundant solar irradiation, with an average of more than 2,000 kWh/m² per year in Ouagadougou^41^. This substantial solar resource underpins the rapid expansion of solar PV capacity and also provides strong justification for the deployment of solar thermal applications, including solar water heating, which can reduce reliance on biomass and imported fuels while meeting household and institutional energy needs.

###  Historical development, local industry and policy framework

Recognising this potential, public authorities implemented early and participated in initiatives to harness solar energy, including solar thermal projects. In 1978, the West African regional centre on solar energy (CRES, Centre Régional de l’Énergie Solaire) was established. This regional institution (11 countries) aimed to train energy specialists and spearhead research and development in solar energy systems^42,43^. This marked Burkina Faso’s initial foray into harnessing solar power to address its energy needs. Unfortunately, the international support expected for by the CRES was not forthcoming, leading to its closure by the end of the 1980s.

**Fig. 5:**
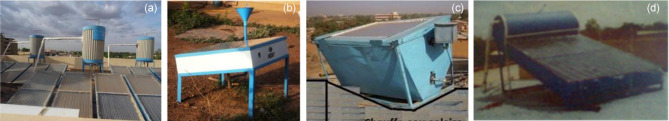
Locally manufactured SWHs in Burkina Faso: (**a**) classic local indirect SWH ATESTA, (**b**) integrated collector storage IRSAT, (**c**) integrated collector storage Actualité Énergie, and (**d**) direct SWH Cité Picasso^8^.

In Burkina Faso, unlike other Community of West African States (CEAO, Communauté Economique de l’Afrique de l’Ouest) member countries, except Mali and Niger, the ambition for local SWH production persisted after the CRES industrial collapse. The State-funded IRSAT (Institute for Research in Applied Sciences and Technologies, founded in 1980) and the Swiss NGO “Centre Écologique Albert Schweitzer” (CEAS) played key roles in maintaining local manufacturing capacity^8,44^. Local craftsmen, trained by CEAS or the Association for the Promotion of Solar Energy (APEES), primarily ensured this production, with small companies operating on a make-to-order basis^8^. By 2015, local manufacturing supplied most of the SWHs in operation in Burkina Faso, all of which were thermosiphon systems (Fig. 5)^8^. Last SWH dissemination programs have prioritised national manufacturers. A 2016 project aimed to install 5,000 SWHs by 2020, focusing on health centres^45^. An earlier project under the Strategy for Accelerated Growth and Sustained Development (SCADD) aimed to install 450 SWHs in health centres using locally manufactured integrated storage collectors (Fig. 5b)^46^.

Since the late 2010s, imported SWHs, especially WiG-ETCs, have dominated the market due to their lower cost than locally manufactured SWHs. New installations of locally manufactured SWHs have practically disappeared, except within public state-supported projects as previously indicated. Local manufacturers struggle with high raw material costs and lack State support for innovation and research. Burkina Faso offers import duties and VAT exemptions for solar equipment, including SWHs^47–49^, but these government support mechanisms mainly benefit importers, leaving local manufacturers at a disadvantage.

###  Main applications

SWHs are primarily installed in households for hot water production. The main use is for bodily hygiene, particularly during periods when temperatures are relatively low, such as during the Harmattan (November to February) and the rainy season (July to September). Notably, a large share of families only used hot water for bodily hygiene in December. In August, when rains are heaviest and there is little sunshine, DHW needs may not be met by SWHs alone, prompting users to resort to additional sources for hot water. The rise of an urban middle class has led to greater adoption of SWHs, and SWHs are predominantly found in urban areas^8^. Conversely, rural communities often rely on wood for hot water, as highlighted by a 2019/2020 study involving over 8,000 residents in rural and peri-urban Burkina Faso, revealing that only 6% of those surveyed were aware of SWH technology^10^.

Collective housing in residential contexts is not yet very developed in Burkina Faso. However, various facilities, such as training centres, boarding schools, and religious centres, have adopted SWHs, employing them in a manner similar to households.

The use of SWHs in hotels is similar to that in households, with demand peaking during the Harmattan season, which coincides not only with the low ambient temperatures but also with the peak tourism season in Burkina Faso^11^.

In health facilities, hot water is essential year-round, primarily for caring for maternity patients, including for postpartum hygiene^8^. Health workers note that the use of hot water for postpartum care is more cultural or psychological than medically recommended. SWH installations in maternity hospitals aim to replace potentially hazardous wood fires used by guests, enhancing safety within health facilities.

Industrial applications of SWHs remain limited in Burkina Faso. For example, HOSCO Hospital in Ouagadougou uses SWHs to preheat water for laundry purposes^50^. There have also been attempts to integrate SWHs into shea butter production processes to enhance sustainability^8^. However, these efforts have faced challenges, notably the long payback period due to the seasonal nature of shea butter production: a SWH needs to be exploited as much as possible year-round to quickly pay back the investment.

### Maintenance and reliability issues

**Fig. 6:**
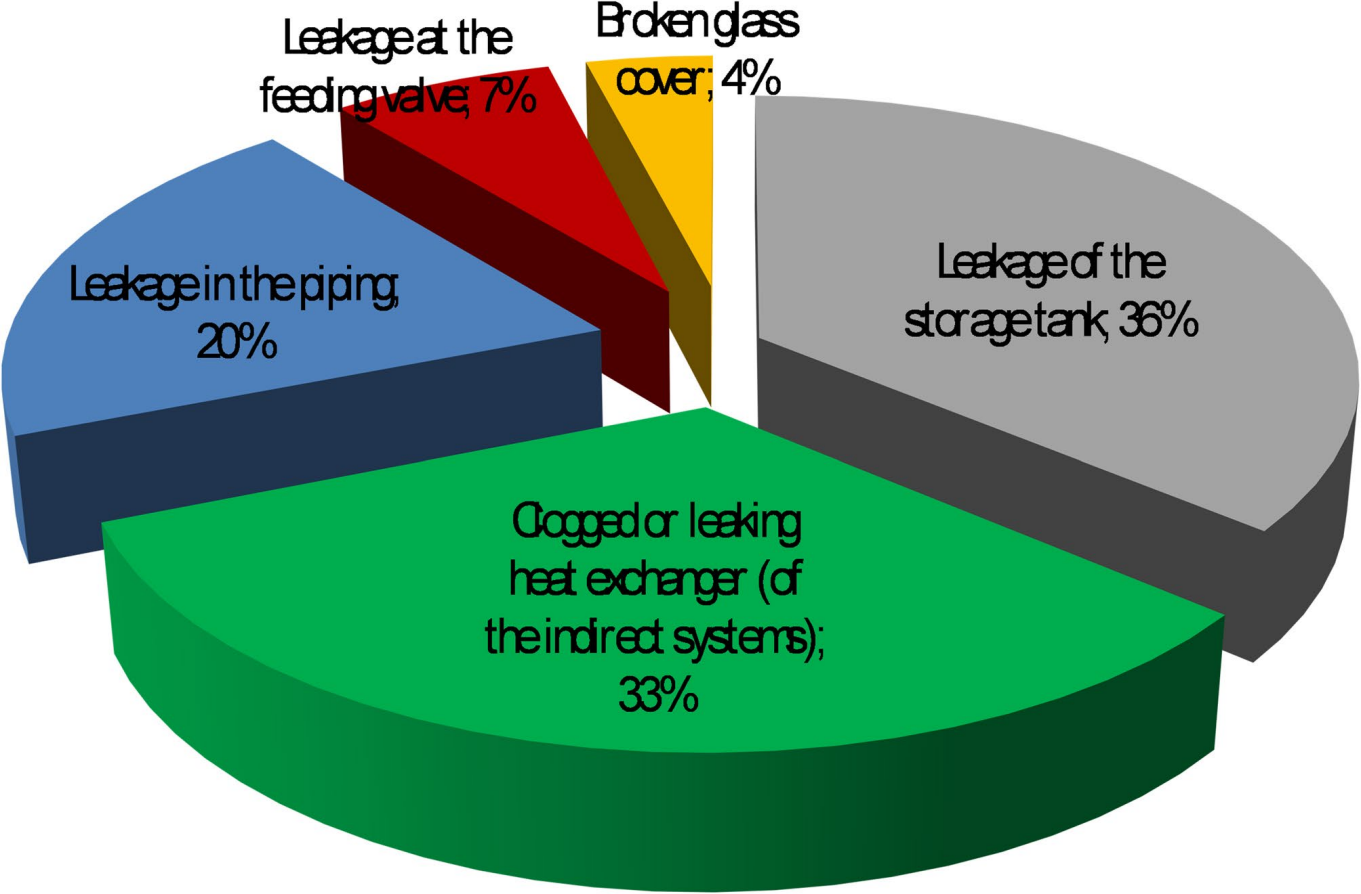
Analysis of failure types in 25 out of service solar water heaters in Ouagadougou based on Bikoï’s 2010 survey^6^.

A study^6^ implemented in 2010 in Ouagadougou revealed that 22% of the 116 surveyed SWHs—predominantly locally manufactured FPC systems installed at the time—were out of service. In 75% of these situations, the cause was maintenance failure; in 24% of cases, the reason was discouragement from repeated failures (Fig. 6). Users suggested that, in addition to potential issues with the competence of maintenance service providers, maintenance challenges may also stem from unfair installation practices and excessive maintenance prices. While these findings reflect an earlier stage of the market, they provide a useful benchmark against which the recent (middle of 2010s) shift toward imported WiG-ETC technologies can be understood.

###  Interest in water-in-glass evacuated tube collector technology in the Burkinabe context

As mentioned earlier, the sales of locally manufactured FPC SWHs and integrated collector storage SWHs have sharply declined, with their market presence now largely limited to government-supported dissemination programs. Consumers have increasingly shifted towards imported systems, predominantly WiG-ETC SWHs. Table 1 outlines the average costs of thermosiphon SWHs and electric heaters in Ouagadougou as of May 2024. For context, the minimum monthly wage is 69 €, while engineers earn an average of approximately 380 € per month.

The costs of industrially imported FPCs with selective coatings are comparable to, and sometimes even lower than, those of locally manufactured FPCs with solar varnish painting. High-performance thermosiphon systems, often meeting rigorous international standards such as those of Solar Keymark, offer superior thermal efficiency and reliability compared to products from local manufacturers. Consequently, local products, which have lost their price competitiveness, are facing near disappearance from the market.

For a given storage capacity, such as 150 L, which is common in household solar water heaters (SWHs), WiG-ETCs are typically half the cost of flat plate collectors (FPCs) (see Table 1), contributing to their greater popularity over FPCs. Indirect WiG-ETCs are priced similarly to direct WiG-ETCs. Stainless steel WiG-ETCs are slightly more expensive than units made from standard steel.

The high cost of tank-based electrical water heaters (accumulators) and the steep electricity charges in Burkina Faso suggest the potential for significant savings through the promotion of SWH.

Most WiG-ETCs available on the market have a fixed frame inclination of 45°. These systems are predominantly imported from China, where solar water heaters (SWHs) are commonly installed with tilt angles above 40° due to the high latitude of 40°^22^. There are, however, a few systems offered with a native tilt angle of 25°. Consequently, without modification, the default inclination for most installed WiG-ETCs in Ouagadougou is 45° when installed on a flat surface. Most of the time, SWHs are installed on tilted roofs, resulting in higher overall tilt angles and sometimes approaching vertical.

According to Fig. 4, the risk of hailfall in Ouagadougou is moderate. In practice, hailfall occurrences in or around Ouagadougou are rare, and only a few hailfall events, such as the event on June 16th, 2007, which occurred 30 km from Ouagadougou, have been of sufficient size to cause significant damage^51^. In the past decade, we have not encountered any instances of SWH system damage due to hailfall.

## Survey of water-in-glass evacuated tube solar water heaters

### Introduction

In Ouagadougou, the capital of Burkina Faso, SWHs have the potential to provide a reliable and eco-friendly alternative to traditional water heating methods. WiG-ETCs have become a key driver in the adoption of SWH technology in the local market. Despite the promising benefits, the actual performance, maintenance requirements, and user satisfaction of these systems under real-world conditions remain under-researched.

This survey aimed to obtain insights into the operational status, maintenance challenges, and user satisfaction of WiG-ETCs for DHW in Ouagadougou. Conducted in April 2024, the survey targeted a diverse sample of households to gather data on the effectiveness and reliability of these solar water heating systems. The findings from this survey will provide valuable insights into the practical performance of WiG-ETCs and inform strategies for improving their adoption and functionality in similar contexts.

### Methodology

#### Survey method

The sample was selected using a purposive and convenience sampling approach. Households with visible WiG-ETC installations used for domestic hot water were identified across various neighbourhoods of Ouagadougou during door-to-door visits in April 2024. When an ETC was identified, the residents were engaged if they agreed to participate. Some participants known to the research team were contacted directly to schedule visits. Households were approached without prior knowledge of whether the systems were direct or indirect; system typology was recorded during the survey, though this was not a primary objective. All WiG-ETCs used for DHW were eligible regardless of system age, condition, or prior usage.

The security challenges in Burkina Faso constrained the number of participants, as many people were reluctant to open doors. The number of households that declined to participate was not systematically recorded due to the door-to-door nature and security context. This situation also limited the collection of detailed demographic and socio-economic data, such as household composition and income, and the refusal rate was not systematically recorded, as the primary focus was on technical system performance and user experience.

**Fig. 7:**
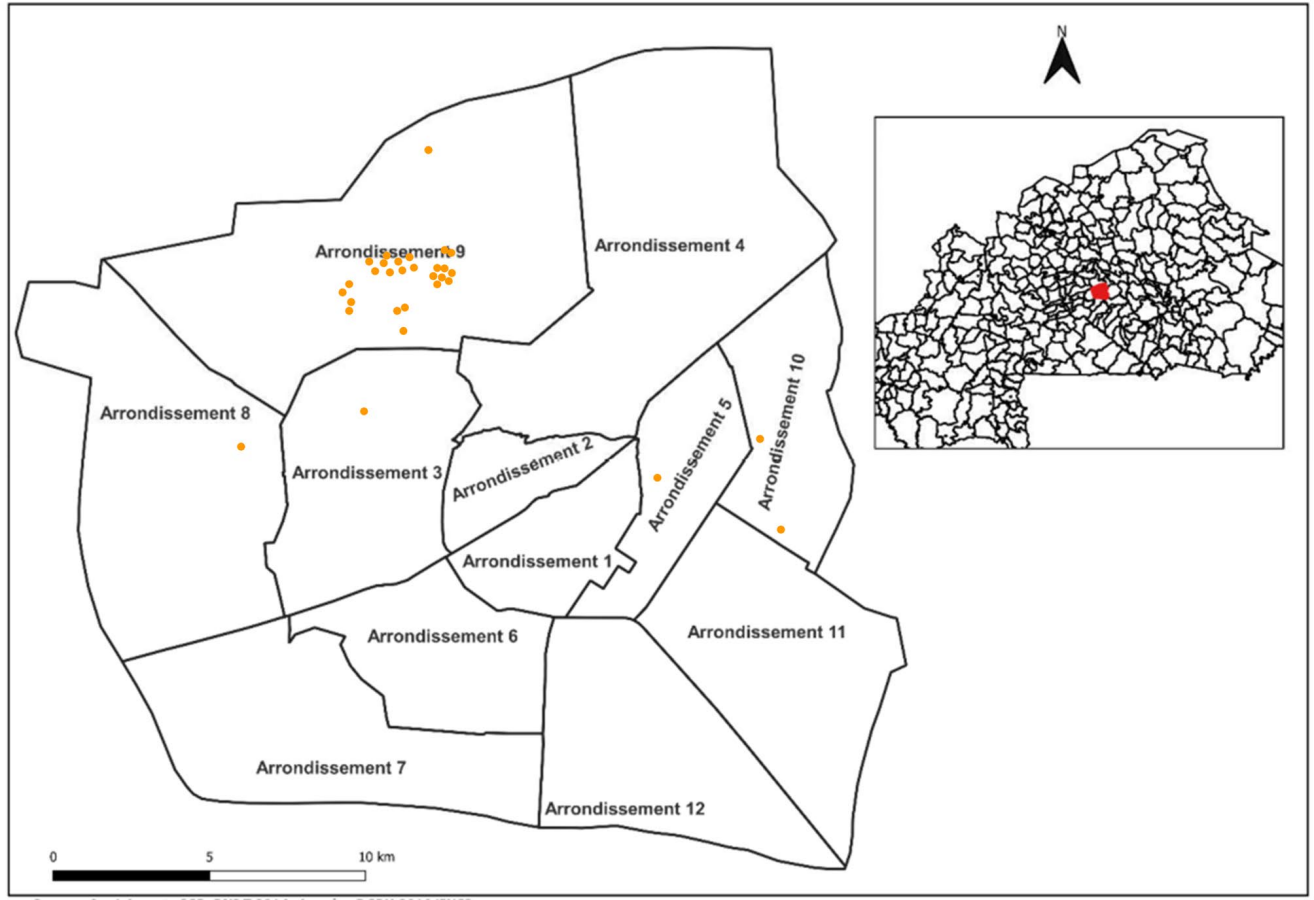
Approximate spatial distribution of the surveyed WiG-ETC systems across arrondissements of Ouagadougou. Base map adapted from Institut Géographique du Burkina Faso^52^.

The approximate geographic distribution of the surveyed systems across the arrondissements of Ouagadougou is shown in Fig. 7. The 31 systems—comprising 24 individual households and a seminary residential facility housing Catholic priests and aspiring seminarians—are predominantly concentrated in Arrondissement 9 (26 systems), with single systems located in Arrondissements 3, 5, 8, 10, and 11. This distribution reflects the purposive door-to-door survey approach and the accessibility constraints imposed by the security context, rather than a systematic sampling of all neighbourhoods in the city. Therefore, the spatial distribution should be interpreted as indicative rather than representative of the overall distribution of WiG-ETC installations in the city.

As a result, the sample is highly concentrated in a single arrondissement (Arrondissement 9) and is based on purposive sampling constrained by accessibility and security conditions. The findings should therefore be interpreted as indicative of conditions within this localised context rather than statistically representative of all WiG-ETC owners in Ouagadougou. Caution is advised when generalising the findings beyond the surveyed households.

This study involved interviews with human participants to collect survey data. No experiments on humans or the use of human tissue samples were conducted. Ethical approval for this research was granted by the 2iE Research Ethics and Deontology Commission. All methods were carried out in accordance with relevant guidelines and regulations, and informed consent was obtained orally from all participants prior to their participation in the study. Participants were informed that the survey was for research purposes, that results would be published, and that their responses would remain confidential and anonymised.

####  Interview questions

The interview questions arise from frequent and dominant themes that are in prior discussions and interventions of the researchers with local users and installers as well as aspects that the researchers consider essential. Participants were asked the following predefined questions:

- For how long has your system been running?
- Have you encountered any breakdown or failure? If yes, which ones?
- Have you already replaced your float valve?
- Have you experienced water leakage at the SWH?
- Are you satisfied with your investment?
- Are your hot water needs met all year-round?
- How many people are using the system? (When possible, specify the number of people above 12 and those at 12 or below).

####  Inspection criteria

A subsequent inspection of the systems included:

- Counting the number of tubes.
- Determining the material of the tank (steel vs. stainless steel).
- Determining the native tilt angle of the collector, which is the tilt angle if it is installed on a horizontal roof.
- Noting any signs of rust on the tank.
- Presence and use of electrical heating element on the WiG-ETC SWH.
- Observing any particularities.

#### Data analysis

A total of 31 systems with sufficient descriptions were included in the analysis. The seminary residential facility hosts 7 separate systems of diverse sizes: 3 with 12 tubes, 1 with 24 tubes, 1 with 30 tubes, and 2 with 36 tubes. The data collected during the survey can be found as Supplementary Table S1 online. Although a larger data pool would be desirable, the collected information provides a sound basis to achieve the study’s objectives. Data analysis was performed using Microsoft Excel for descriptive statistics and comparative calculations.

### Results and discussion

The responses are analysed below, focusing on system functionality, maintenance issues, user satisfaction, and the overall performance of the WiG-ETC SWHs, with quantitative metrics included. As presented in Sect.  4.2, the survey collected two distinct types of data: directly observed technical parameters (tube count, tank material, tilt angle, rust presence) and user-reported information (system age, failure history, satisfaction, year-round hot water availability). No continuous performance monitoring or direct temperature measurements were conducted.

#### Trend of technology adoption and user satisfaction

**Fig. 8:**
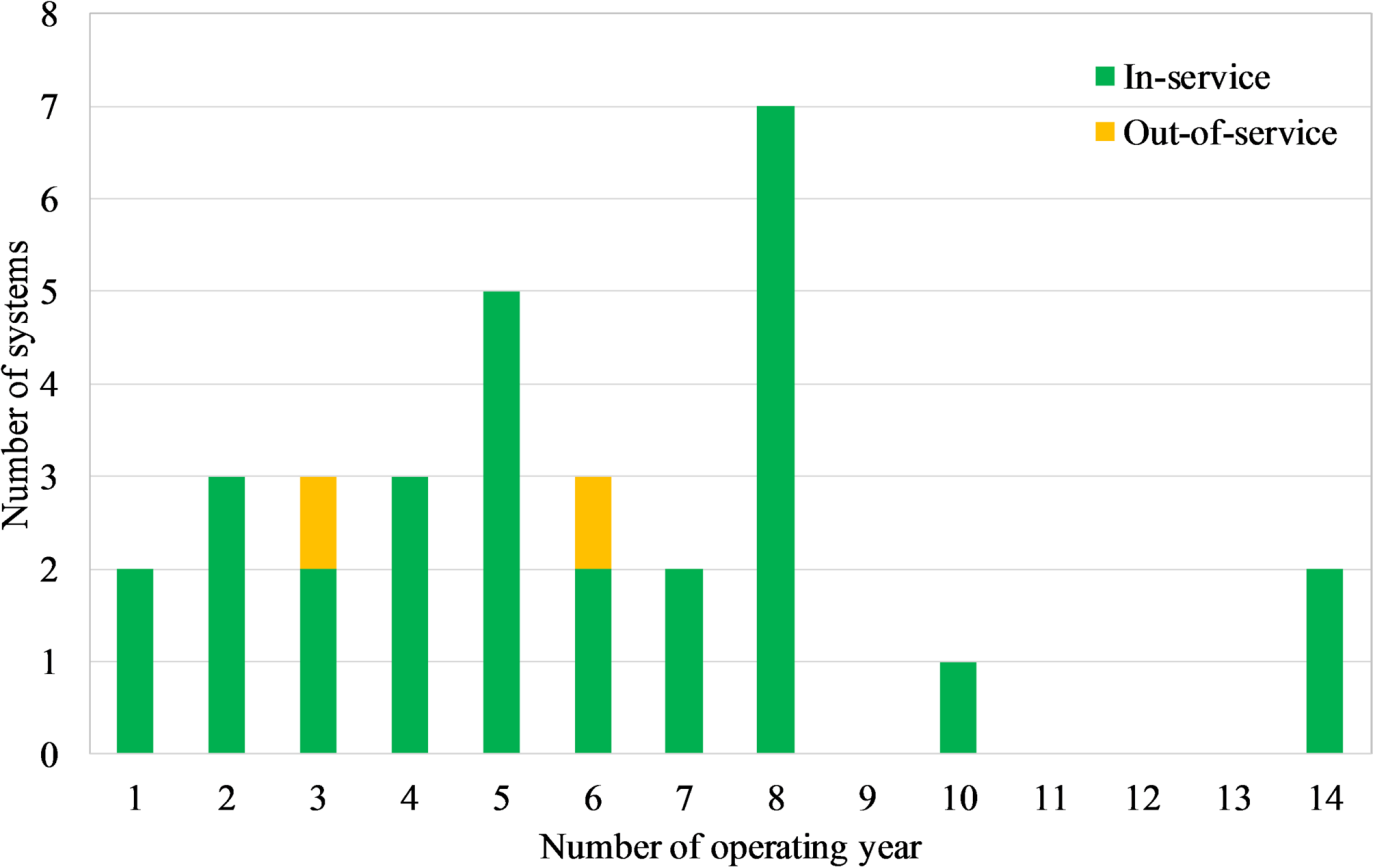
Distribution of the number of systems based on the age of the WiG-ETC SWHs.

90% of SWHs have been installed in the last 8 years (Fig. 8). Noting that the 7 SWHs that are 8 years old correspond to the seminary residential facility, the data show that yearly installations range from two to five systems (Fig. 8) over the past 7 years, with no sharp peaks but a regular introduction of systems each year. This indicates a sustained diffusion of WiG-ETCs across the surveyed sites, although the small sample size (31 systems) limits the extent to which long-term market trends can be generalised.

**Fig. 9:**
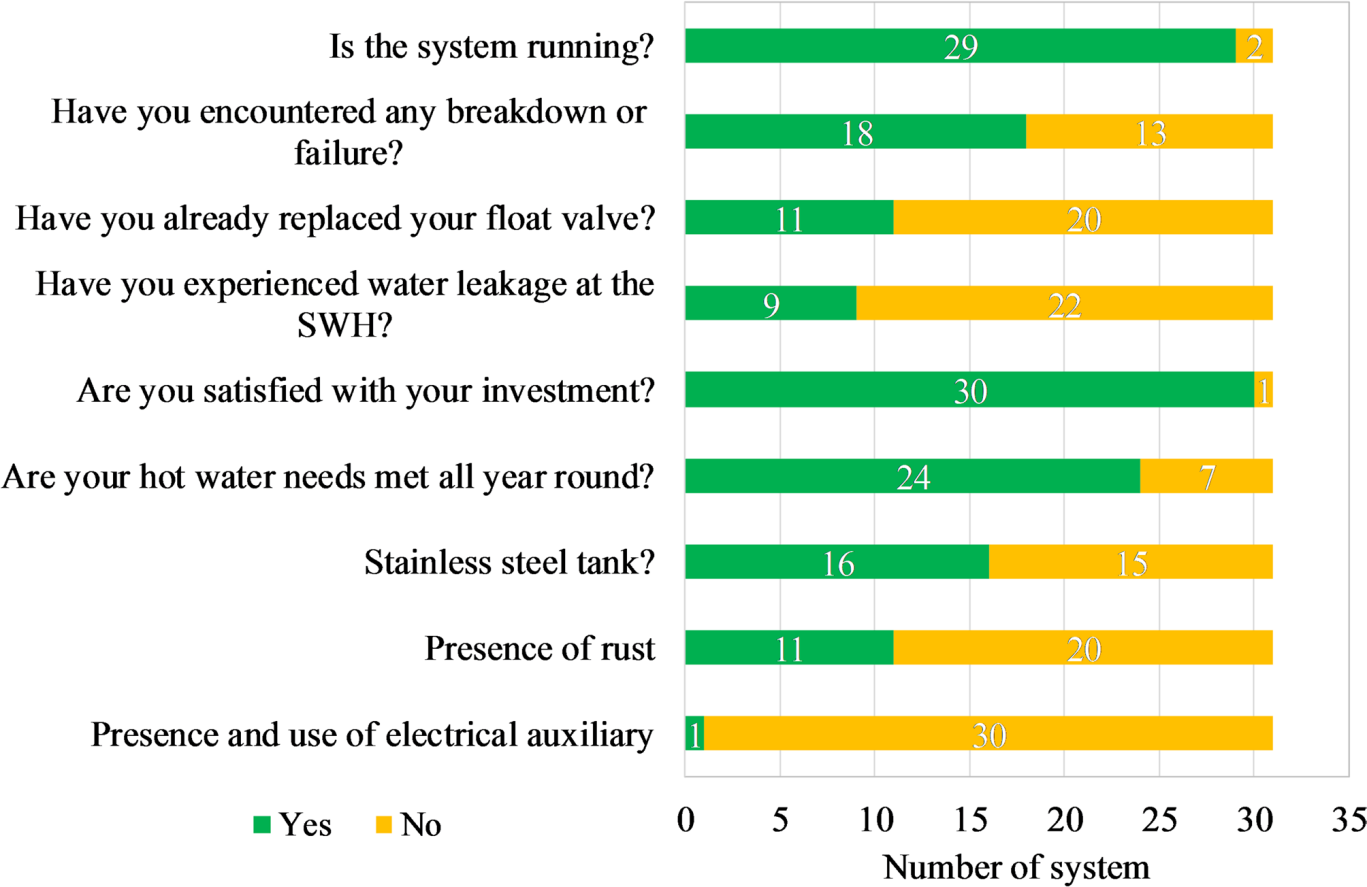
Operational status, maintenance issues, and user satisfaction of the WiG-ETC SWHs.

The consistent installation trend reflects a growing adoption of WiG-ETCs in Burkina Faso. This trend is supported by a very high level of user-reported satisfaction (97%), despite prevalent maintenance challenges and occasional system failures (Fig. 9). The sustained installation trend may be further driven by increasing familiarity and positive word-of-mouth within communities, factors known to catalyse the adoption of solar technologies^53^. This is supported by our field observations, which noted a clustering of WiG-ETC installations within specific neighbourhoods, suggesting a peer-influenced diffusion pattern. It should be noted, however, that these observations are based on cross-sectional data collected at a single point in time. Longitudinal monitoring would be required to confirm whether current user satisfaction and installation trends persist over the long term. The apparent contrast between frequent technical issues and near-unanimous satisfaction suggests that users prioritise the availability of hot water, convenience, and strong thermal performance over perfect operational reliability. This finding contrasts with studies of subsidized SWH programs, where satisfaction is often compromised by a gap between promised and delivered benefits. For example, research on a government-led rollout in South Africa found a statistically strong negative relationship between user expectations and satisfaction, indicating that inflated promises can lead to disappointment even when systems are functional^1^. In the consumer market of Ouagadougou, expectations for WiG-ETCs appear to be pragmatically shaped by their affordable price point and observed performance within the community. The technology’s ability to reliably meet the core expectation of providing domestic hot water for most of the year seems to generate a strong positive disconfirmation, outweighing the noted maintenance nuisances.

This situation also differs markedly from the historical period in Burkina Faso when locally manufactured flat plate collectors (FPC SWHs) dominated the market and faced scepticism due to reliability issues^6^ but also from the experience of structured government rollouts elsewhere in Africa. For instance, an assessment of the national Solar Water Heating Programme in South Africa revealed systemic failure, with a majority of installed systems becoming non-functional due to poor-quality installations, lack of maintenance, and damage from environmental factors like hail, leading to widespread user dissatisfaction and program abandonment^29^. In turn, positive user experiences and perceptions can lead to increased adoption rates among households and businesses seeking reliable, cost-effective solutions for hot water provision, while providing policymakers with justification to implement supportive incentives for wider deployment.

This underscores that for market-driven adoption, the primary challenge may not be managing abstract expectations, but rather ensuring the long-term hardware durability and serviceability that underpin continued positive experience.

#### Operational reliability and critical failure modes

Among the 31 systems surveyed, 94% were operational at the time of the survey (Fig. 9). Common issues included malfunctioning float valves (35%) and water leakage (29%), with 13% of systems indicating both types of failure. Notably, 8 of the 9 systems reporting water leakage had been in operation for at least 5 years (Table 3). These statistics indicate that, while most WiG-ETC SWHs are functional, certain components become increasingly prone to failure with system age.

**Table 3:** Distribution of reported failures by system age.

Failure type	Systems < 5 years old	Systems ≥ 5 years old
Water leakage	1	8
Float valve	2	9
Any (including both above)	3	15

The most frequently reported issue is related to the float valve (Fig. 9), which is located inside the assistant tank, and affects both stainless steel and standard steel systems. Several mechanisms may contribute to these failures. The clearest observed pattern is an association with system age: most failures occurred in systems that had been in operation for at least five years (Table 3), suggesting that corrosion, material fatigue and gradual wear are likely contributing factors to malfunction. However, as the analysis is based on cross-sectional observations, causal relationships cannot be definitively established.

Environmental exposure is also likely to play a significant role. Ouagadougou, like much of the Sahel, is strongly affected by airborne mineral dust originating from the nearby Sahara Desert—responsible for more than half of the global atmospheric dust load^54,55^. The assistant tank is vented to the atmosphere through an open pipe, typically oriented upward, providing a direct pathway for dust ingress. Over years of operation, fine particulate matter may accumulate around the float mechanism, accelerating abrasion or causing intermittent obstruction. These dust effects combine with high ambient temperatures and strong diurnal temperature fluctuations, characteristic of the Sahel, which may further stress materials and seals.

Debris and sand from the water network, particularly after shortages or pipe maintenance, may also clog or erode the fill valve and contribute to malfunction. However, this appears to be a secondary factor compared with ageing and environmental exposure. This finding is consistent with earlier work in Ouagadougou (2010), which already reported clogging as a major source of SWH failure^6^.

**Fig. 10:**
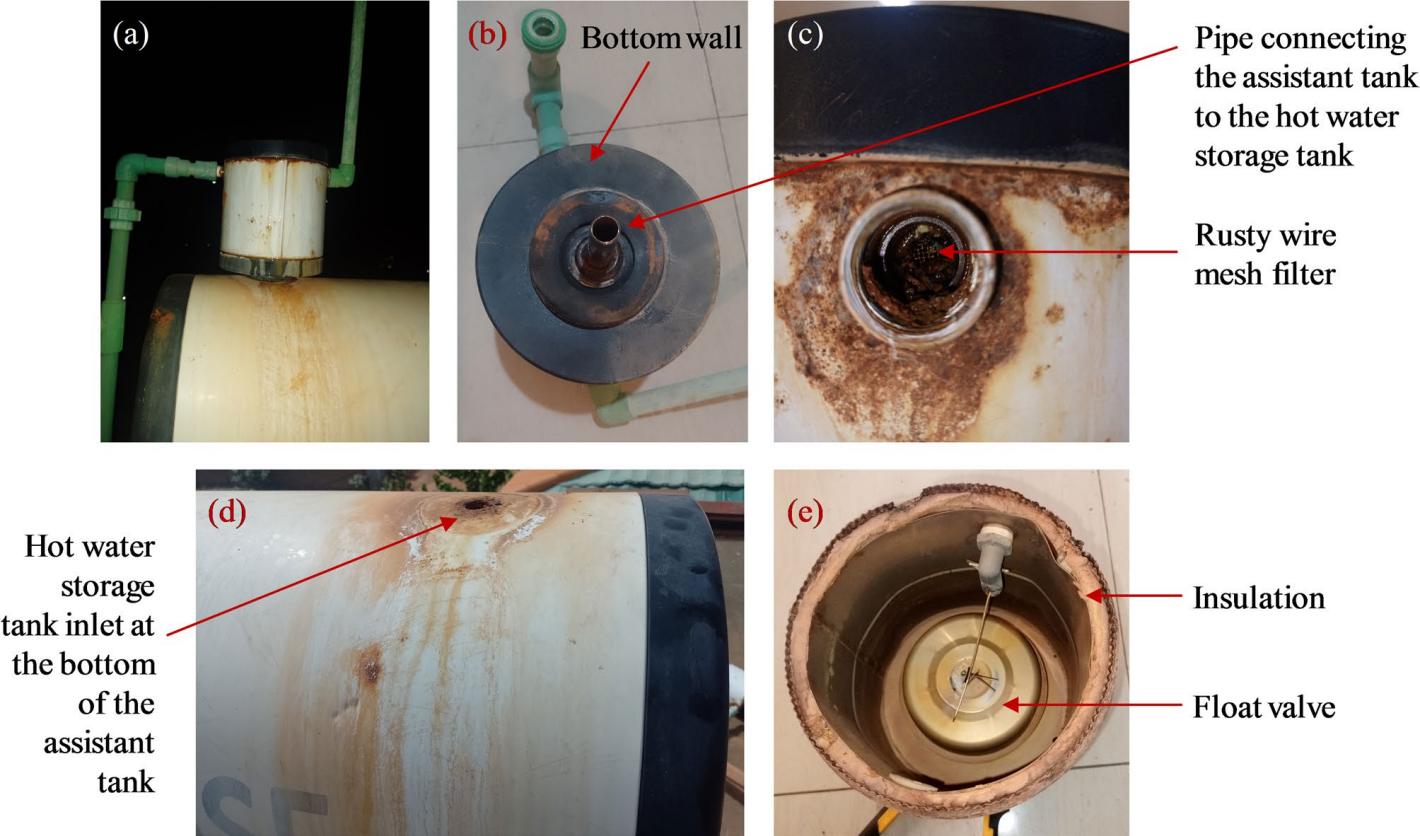
Assistant tank of the system out of service after 6 years: (**a**) leaking assistant tank, (**b**) bottom view and (**c**) cold water inlet orifice, (**d**) rusty hot water storage tank and (**e**) opened assistant tank. The system was still in operation one month before the survey.

The disassembly of one out-of-service system revealed a rusted and partially clogged wire-mesh filter at the inlet of the assistant tank (Fig. 10). The rust is most likely a symptom of long-term contact with water and progressive material ageing, although it may, in turn, worsen inlet obstruction. Since public utility water in Ouagadougou—which feeds most SWHs—is not typically hard, scaling is unlikely to be a primary cause. Other contextual stressors—such as strong pressure fluctuations in the water network and variable material quality—may interact with the mechanisms described above. Further investigation is needed to determine the relative contribution of each factor.

These findings point to several potential design adaptations. Dust-related degradation could be mitigated through vent reorientation or protective screening, while corrosion could be reduced through improved material selection or protective coatings for float assemblies and internal fittings. Additionally, because most assistant tanks are sealed units, maintenance is difficult; adopting a more maintainable design with an openable interior would facilitate inspection and repair. We observed this advantage in one locally adapted installation that used a custom-designed, openable assistant tank.

Another prevalent issue reported in the survey is water leakage, often associated with float valve failures but not limited to them. Leakage is typically observed at the junction between the assistant tank and the water storage tank, primarily due to sealing failure from aging or corrosion. Additional leakage locations include the top of the assistant tank (Fig. 10a), the junction between the tank and the evacuated tubes, and the main water tank itself. Indeed, on some systems, it is observed that occasionally, hot water can flow back from the hot water storage tank to the assistant tank, causing several litres of water to overflow from the assistant tank. Leakage observed in some tanks—indicating that it had penetrated the insulation and caused internal damage to the tank— result from holes in the tank wall, potentially caused by long-term superheating due to low consumption during sunny periods. At the junction between the tank and the evacuated tubes, leakage can result from washer failure. This is a documented failure mode where seals degrade under high operational temperatures and thermal cycling^31^. However, while studies in regions with hard water in India attribute such failures partly to abrasive friction with salt crystals^31^, the primary cause in Ouagadougou is more likely related to material quality and extreme environmental exposure, given the city’s relatively soft water supply^31^. In one system, after replacing some broken pipes, glass debris from the broken tubes remained in the tank and may have deteriorated the washer. However, replacing the washer did not resolve this issue.

The high frequency of these failures has significant economic implications. Temporary fixes, such as using Teflon tape, are not permanent solutions. The core of the problem lies in the scarcity and high cost of replacement parts on the local market. Indeed, the cost of an assistant tank, as of May 2024, ranges from 53 € (standard steel) to 72 € (stainless steel) (Table 4), representing 15–20% of the initial system cost for popular WiG-ETC systems (100–150 L units). Washers are also often unavailable on the local market and are often obtained through recuperation, making them expensive (up to 15 € per piece, Table 4).

**Table 4:** Cost of critical replacement components on the local market in Ouagadougou (May 2024), illustrating the significant economic burden of maintenance.

Component	Price [€]
Assistant tank made of standard steel	53
Assistant tank made of stainless steel	72
Evacuated tube	12–30
Washer ^a^	15

^a^ Usually, unavailable on the local market.

This high cost of maintenance imposes a substantial economic burden on users and is a critical factor that must be considered in any assessment of the long-term economic viability of WiG-ETC SWHs. The critical importance of proactive seal inspection and leakage control to prevent cascading failures has been emphasized in technical failure analyses^31^. Our findings reinforce that adopting such preventive maintenance practices is essential in the Burkinabe context to preserve the longevity of WiG-ETC investments. Consequently, ensuring the local availability of high-quality, durable components is essential for reducing downtime, improving system reliability, and safeguarding user satisfaction.

Minor failures, such as pipe breakages were rare and generally resulted from mechanical shocks rather than blockages or salt formation in the tubes, contrasting with findings elsewhere^31^. The water quality in Ouagadougou appears adequate to prevent significant salt formation and limestone deposits. However, for systems using borehole water, attention should be given to water quality to avoid potential issues.

####  Material durability and system longevity

Two systems were out of service at the time of the survey, having operated for 3 and 6 years, respectively (Fig. 8). Both were not made of stainless steel. The system that lasted only three years (2015–2018) broke down, according to the owner, due to a float valve failure. The repair could not be completed because the plumber took the defective float valve and never returned and replacement parts were difficult to obtain locally. The tank now exhibits significant rust. The second system, operating from 2018 to 2024, experienced extensive leakage affecting the storage tank, the inner tank, the outer protective layer, and the assistant tank (Fig. 10). Since it is not possible to purchase only the storage tank locally, the owner plans to replace the entire system with a stainless steel version. This corrosion susceptibility was widespread; indeed, 11 of the 15 non-stainless steel systems showed signs of rust (Fig. 9), highlighting the high susceptibility of standard steel tanks to corrosion.

Approximately half of the surveyed systems are made of stainless steel (Fig. 9), including the two oldest systems, which have been operating for more than 14 years (Fig. 8). However, a 10-year-old system standard steel system exhibited no rust, although its owner reported replacing the float valve and experiencing water leakage.

**Fig. 11:**
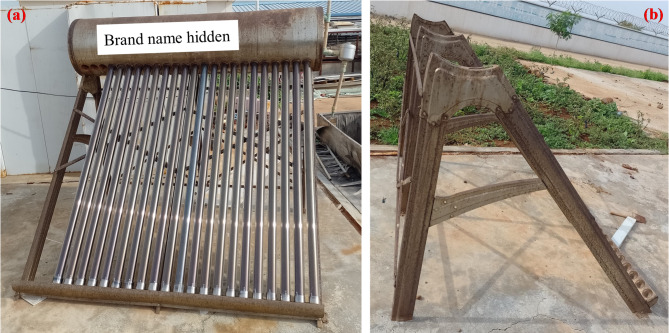
(**a**) WiG-ETC made of stainless steel out of service and (**b**) with a frame that underwent corrosion due to proximity to the sea (this picture is not from Ouagadougou but from Praia, Cabo Verde).

These observations provide insight into the potential life expectancy of these systems under local conditions. The oldest stainless steel systems in the survey had been operating for more than 14 years and remained functional, indicating that a service life of at least this duration is attainable under the surveyed conditions, with minimal tank-related issues aside from periodic assistant tank replacement (due to float valve failure). In contrast, most standard steel tanks observed had developed significant corrosion within approximately 6 years, with one system having failed entirely. However, as these observations are derived from a single survey snapshot, they should not be interpreted as definitive estimates of expected service life without further longitudinal validation. Given the relatively small cost difference between stainless steel and standard steel tanks (Table 1), prioritising stainless steel is advisable. However, the quality of stainless steel must match environmental conditions: in coastal Sahelian countries, we have encountered some stainless steel WiG-ETC installations that have corroded due to high air salinity (Fig. 11). Similar behaviour has been reported on SWHs in coastal Taiwan, where standard grades (e.g., 304) are prone to corrosion whereas higher-grade alloys such as 316 exhibit significantly better durability^56^.

#### System sizing, installation practices, and local adaptations

**Fig. 12:**
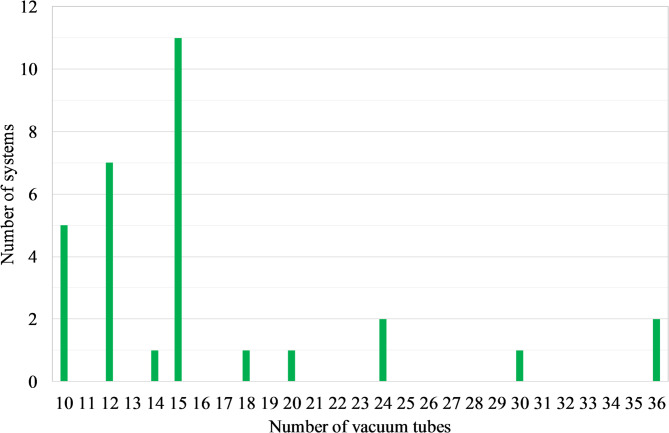
Distribution of the number of systems based on the size of SWHs.

**Fig. 13:**
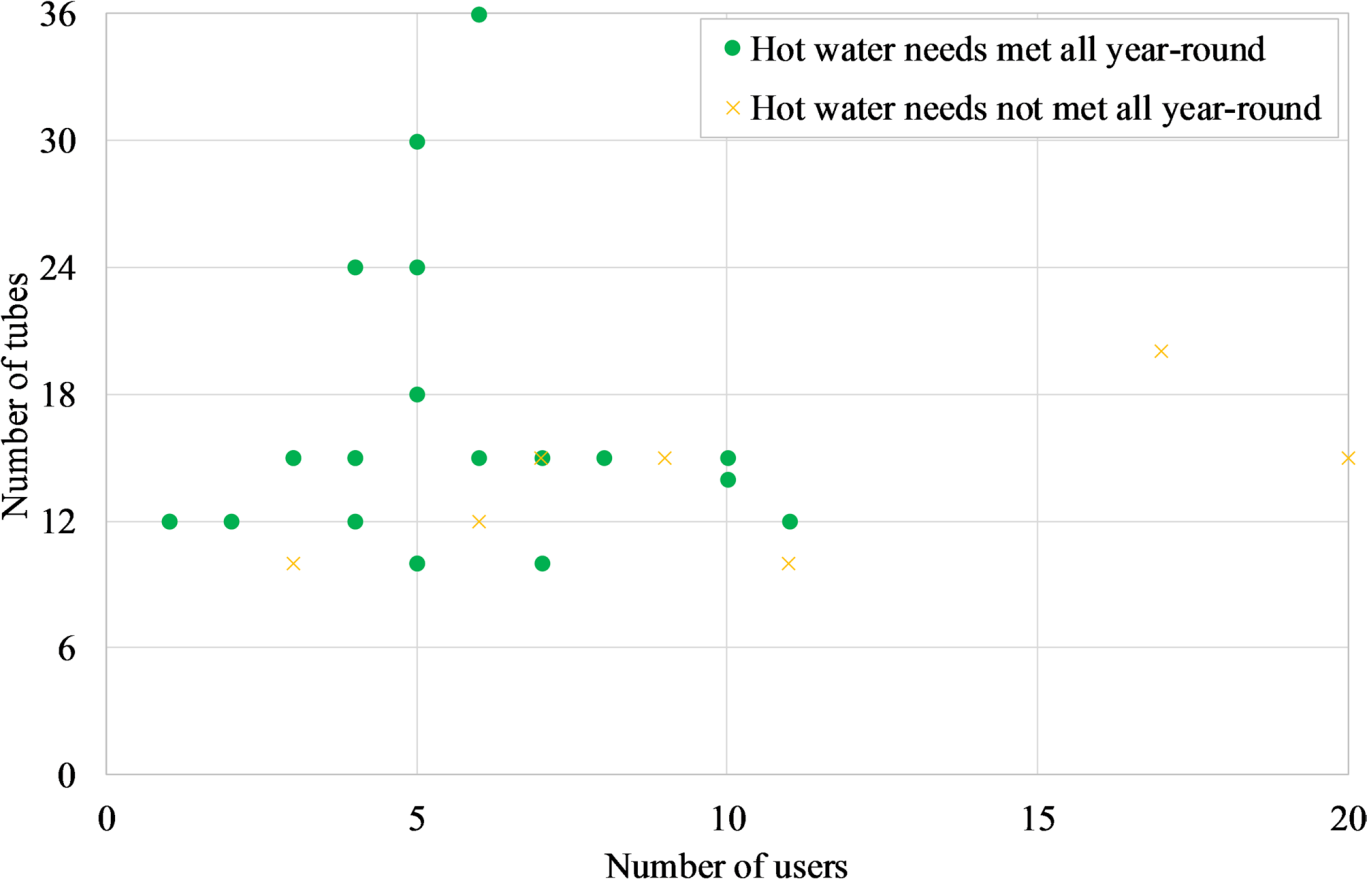
Distribution of users by size of SWH.

The most popular system size is the 15-tube SWH (Fig. 12), which typically corresponds to a capacity of 150 L. This size seems generally sufficient to meet the hot water needs of households with up to 8 or even 10 people but is likely inadequate for households of 20 people (Fig. 13). Regarding hot water availability, 74% of the owners reported that their hot water needs were met throughout the year. This corresponds with the very high share of them (97%) who reported being satisfied with their investment (Fig. 9). The only dissatisfied user operates an undersized system (10 tubes for 11 people).

To further examine seasonal adequacy during the coldest period (December–January), when hot water demand is typically highest in Ouagadougou, a complementary follow-up was conducted on two households equipped with 150 L WiG-ETC systems. These households included 7 (3 adults and 4 children including 3 teenagers) and 9 (3 adults and 6 children including 3 teenagers) occupants, respectively. From early December 2025 to the end of January 2026, weekly feedback was collected on whether hot water needs were fully met. One of the monitored households belonged to a co-author; however, only binary operational feedback (sufficient/insufficient) was recorded to limit subjective interpretation. Both households consistently reported sufficient hot water throughout the monitoring period. Although this qualitative follow-up does not constitute controlled performance measurement, it supports the possibility that 150 L WiG-ETC systems may be sufficient to meet peak seasonal demand under typical local conditions.

These results suggest that the 15-tube system is well-adapted to the majority of households, balancing capacity, cost, and practical usability. They indicate a derived hot water demand in the local context of approximately 15 to 19 L per person per day, which is significantly below typical developed-world sizing guidelines of 35–50 L per person per day (at 50 °C) commonly used for SWH design^57^. This discrepancy highlights the importance of context-specific demand assessment and warrants further investigation into daily usage patterns and the influence of climate on hot water needs in Sahelian households.

Almost all the surveyed SWHs (94%) have a native tilt angle of 45°, while the remaining 6% (2 SWHs) have a native tilt angle of about 25°. Both of the latter are of standard steel systems, aged 3 and 4 years.

**Fig. 14:**
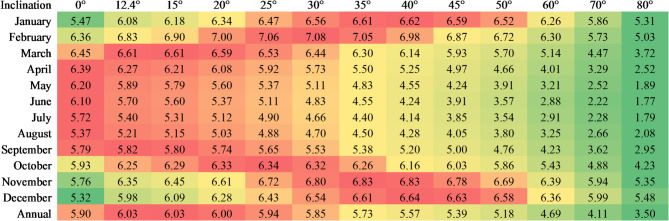
Average daily solar irradiation [kWh·m^-2^·day^-1^] as a function of month and surface inclination. Data obtained from RETScreen computations for Ouagadougou, based on monthly averages of solar radiation (1961‒1990). A colour scale is applied for each month (row) to facilitate comparison.

Hence, most systems have a relatively high collector tilt, with a minimum of 45°, which is the default inclination for WiG-ETC frames available locally when installed on flat surfaces. Provided that necessary measures are taken to ensure the system’s stability, frames can be adjusted for optimal tilt, but this is rarely done, and the native angle is typically used. An inclination of 45° results in approximately 10% less solar irradiation on the collector in Ouagadougou (latitude 12.4°) (Fig. 14). However, this angle may be appropriate depending on the consumption profile: for households with peak hot water demand during the Harmattan season (November–January, the local “winter”), which is the case in Ouagadougou^9^, a 40–45° tilt is suitable. In contrast, systems aiming to maximise year-round solar gain, such as a hospital laundry SWH^50^, would benefit from an inclination closer to the latitude (~ 15°). This remains a hypothesis requiring empirical validation, as previous studies^22,33,34^ indicating the minimal effect of inclination angle on WiG-ETC performance have been conducted for higher angles (22° to 45°) and latitudes. Other potential limitations, such as hydrodynamics and a significant share of the volume of water below the tube level in the tank (φ ≈ 34%) (Fig. 3), may become prominent at lower inclinations. Further investigations are needed to determine the energy gain of systems installed on tilted roofs, which results in higher tilt angles, such as 60°–70°.

As summarised in “Introduction to water-in-glass evacuated tube collectors”, direct and indirect WiG-ETCs differ significantly in terms of pressure requirements and water stagnation risks (Table 2). All ETCs surveyed were direct WiG-ETCs. Beyond economic considerations—direct systems are cheaper than indirect ones (Table 1)—their popularity can be attributed to their ability to store DHW. Public water supply services in Burkina Faso, including in Ouagadougou, often experience periods of low pressure that are insufficient to supply rooftop SWHs. Water supply interruptions (shortages) also occur during certain periods of the year. Under such conditions, indirect WiG-ETCs become difficult to operate when pressure is low, water cannot be pushed through the internal heat exchanger or even reach rooftop systems. Although a pressurising device could address low pressure, it remains ineffective during complete water shortages. Moreover, such pumps depend on a stable electricity supply—a significant vulnerability in Burkina Faso, where the System Average Interruption Duration Index (SAIDI) reached 358 h in the most recent report^18^, a figure generally representative of conditions in Ouagadougou^19^. Direct systems, by storing water in the tank, avoid this dependency entirely.

By contrast, direct WiG-ETCs store domestic hot water (DHW) in the tank itself and continue to provide hot water during shortages. Tanks typically refill overnight when pressure recovers. This operational resilience is probably a major driver of user satisfaction and helps maintain sustained adoption, as uninterrupted access to hot water is perceived as a key indicator of system reliability.

These advantages come at the expense of increased stagnation risk, whereas indirect systems offer better control of water quality and minimise microbial growth. However, a study in 2024^20^ found no pathogenic legionella (*Legionella pneumophila*) in SWHs in Ouagadougou, and the disease is not reported in local health systems. Direct systems nonetheless require periodic cleaning and adequate tank protection to maintain long-term water quality.

An additional contextual factor may further explain the dominance of direct WiG-ETCs. Locally manufactured FPCs are predominantly indirect systems because this configuration avoids exposing the black-steel storage tank to raw water. Instead, a galvanised steel or copper heat exchanger operates within a closed, pressurised loop to limit corrosion and water-quality degradation. The technical evaluation conducted in 2010 reported that approximately 33% of failures were due to blocked or perforated heat exchangers—failures directly associated with indirect configurations in a low-pressure, dust-laden environment. Compared with these earlier experiences, the direct configuration of WiG-ETCs avoids exchanger-related vulnerabilities and is therefore better suited to local hydraulic and environmental constraints.

**Fig. 15:**
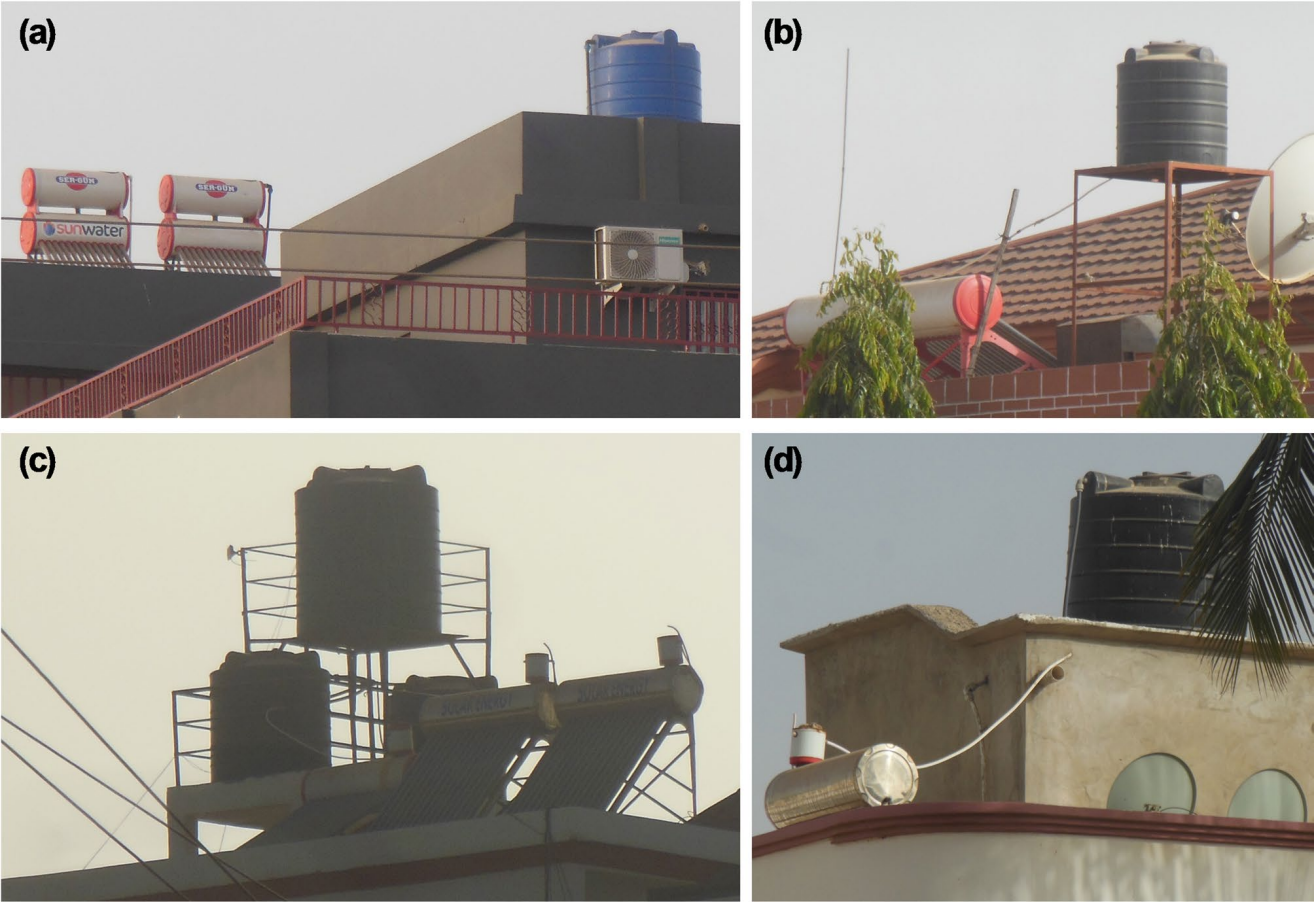
Evacuated tube collectors in Serekunda, The Gambia: Configuration (**a**) features cold water storage in an integrated tank to ensure continuous operation during water service interruptions. Configurations (**b**), (**c**), and (**d**) utilise a separate storage tank for water supply.

To mitigate pressure-related challenges, an alternative configuration—widely adopted in The Gambia—integrates a dedicated cold-water storage tank, either separate or built into the WiG-ETC design. This solution ensures continuous operation during shortages and represents a viable option for Ouagadougou’s context as well (Fig. 15).

###  Limitations

This study has several limitations, primarily stemming from the challenging local context. The foremost constraint was the security situation in Burkina Faso, which necessitated a purposive and convenience-based sampling approach for safety and practicality. Households were selected specifically because they had visible WiG-ETC installations (purposive) and were accessible/willing to participate (convenience). This method, while appropriate under the circumstances, means the sample of 31 systems may not be fully representative of all WiG-ETC users in Ouagadougou, potentially introducing selection bias. For instance, it may underrepresent households in less accessible neighbourhoods or those who were reluctant to engage due to security concerns. Consequently, the findings on adoption trends and satisfaction should be interpreted as strong indicators rather than definitive population-wide statistics.

Furthermore, the study relies mostly on self-reported data, which is subject to recall bias. This is particularly relevant for seasonal hot water availability, as no direct measurements of water temperature or thermal output were conducted across different seasons. Future studies should complement user surveys with seasonal performance monitoring to validate these perceptions. In addition, the survey was conducted in a single month (April 2024), and system performance and user satisfaction may vary throughout the year due to seasonal changes in weather and hot water demand.

Water supply source was recorded for only 15 of the 31 systems (the seminary facility with 7 systems using borehole water, and 8 households using public utility water). For the remaining 16 households, this information was not collected. This represents a missed opportunity, as water quality can vary between sources and may influence corrosion rates and component degradation. Because water quality parameters were not systematically measured, the observed relationships between system age and component degradation cannot be attributed to specific water quality effects. Interpretations of corrosion patterns should therefore be framed as associations rather than causal relationships. However, it is worth noting that in the Ouagadougou context, the vast majority of households rely on public utility water, so the unrecorded cases are likely consistent with the 8 households where public utility use was confirmed. Nevertheless, future studies should prioritise systematic documentation of water source and, ideally, direct measurement of water quality parameters to enable more rigorous causal analysis.

Finally, the survey did not gather detailed information on the frequency of failures or component replacements, so multiple interventions on the same system may be considered single.

## Conclusion, practical implications and outlook

### Summary of key findings

In Ouagadougou, Burkina Faso, water-in-glass evacuated tube solar water heaters (WiG-ETCs) have emerged as a vital technology, progressively replacing traditional heating methods due to their compelling affordability, reliability, and high user satisfaction. This study provides a foundational analysis of WiG-ETC field performance, maintenance challenges, and user satisfaction within this unique socioenvironmental context.

The survey revealed a consistent trend toward adoption of WiG-ETCs, with 90% of surveyed installations occurring in the last eight years. This growth is underpinned by a 97% user-reported satisfaction rate, a significant positive shift compared with past scepticism towards solar thermal technology. However, this satisfaction coexists with prevalent maintenance issues. Float valve failure (35% of systems) and water leakage (29%) are identified as the primary technical bottlenecks, driven by environmental factors and the corrosion of standard steel components. Because these findings are derived from a single cross-sectional survey, they reflect conditions observed at a specific point in time and should be interpreted as indicative of trends and associations rather than definitive causal relationships or long-term performance guarantees.

The study also clarifies two important design considerations. First, the prevalent 45° installation tilt, while suboptimal for annual solar gain, is pragmatically suited to maximising energy collection during the high-demand Harmattan season. Second, the dominance of direct (open-loop) systems is not only an economic choice but a rational adaptation to Ouagadougou’s intermittent water supply, providing a crucial storage buffer that indirect systems cannot.

### Practical implications for key stakeholders

#### For users and system owners

Based on the field results, 150-L WiG systems (≈ 15 tubes) were observed to be adequate for households of 8–10 persons under local usage patterns during the survey period and under reported usage conditions. This finding is based on user-reported adequacy rather than measured consumption and should be considered indicative of typical experience rather than a guarantee. Stainless steel tanks should be prioritised over standard steel, as evidence shows they extend system lifespan from under 6 years to over 14 years with minimal corrosion. While initial costs may be slightly higher, their superior resistance to corrosion and wear justifies the investment. Hence, stainless-steel tanks are recommended to improve lifecycle durability.

#### For technicians and installers

Improved sealing and serviceability of assistant tanks, including protected venting and accessible interiors, are needed to mitigate float valve failures. A tilt angle around 45° appears to be a practical compromise for seasonal demand conditions.

#### For policymakers and market stakeholders

Strengthening quality standards for imported systems and improving local availability of critical spare parts, such as valves and washers, could significantly enhance system reliability, reduce maintenance costs and downtime, and safeguard the technology’s economic viability for users. Looking forward, sustaining this positive adoption trajectory requires concerted action. Policymakers and industry stakeholders can leverage these insights by strengthening the existing market, promoting quality standards for imported systems, supporting local supply chains for components, and considering targeted incentives for durable, serviceable designs.

While imported WiG-ETCs have become the dominant option in Ouagadougou, their widespread use should not overshadow the strategic need to revitalise the local solar thermal manufacturing industry. Relying solely on imports limits local maintenance expertise and reduces opportunities for job creation and industrial development. Although WiG-ETC manufacturing remains challenging at present, local production of FPC systems has existed for decades and could be strengthened. Improving design robustness, better tailoring systems to user preferences, and aligning raw-material tax policies with those granted to imported SWHs would enhance product quality and increase user satisfaction.

### Outlook and future research

The finding that 150-litre WiG-ETCs could adequately serve households of 8–10 persons challenges conventional sizing assumptions and deserves deeper investigation. Future research should examine the long-term stability of WiG-ETC performance under the region’s highly variable environmental conditions. In particular, the root causes of float-valve failures and the performance implications of very high tilt angles remain insufficiently understood. Clarifying these aspects will be essential for optimising system design and maximising energy capture across diverse installation settings. Furthermore, the influence of water quality—especially borehole water—on component degradation and overall system reliability requires systematic assessment. Exploring alternative materials and design improvements to reduce recurrent maintenance issues represents another key area for future work.

## Supplementary Information

Below is the link to the electronic supplementary material.

Supplementary Material 1

## Data Availability

The authors declare that the data supporting the findings of this study are available as Supplementary Information (Table S1) online.

## Abbreviations

ETC: Evacuated tube collector
FPC: Flat plate collector
ICS: Integrated collector-storage
SWH: Solar water heater
SWHS: Solar water heating system
WiG-ETC: Water-in-glass evacuated tube collector
