# Supplementary material for: Field survey insights and performance assessment of water-in-glass evacuated tube solar water heaters in Burkina Faso
Source: Sci Rep. 2026 Apr 11;16:17060. doi: 10.1038/s41598-026-48447-w (PMC13230855; doi:10.1038/s41598-026-48447-w)
Supplement: Supplementary file 1 — Supplementary Material 1 [file 41598_2026_48447_MOESM1_ESM.docx]

# Supplementary Table S1. Data collected during the survey.

| N° | Years | Failure? | Status | Float failure? | Water leakage failure? | Satisfied with investment? | Availability of hot water year-round? | Number of users | Number of tubes | Native tilt angle [°] | Stainless steel? | Rust present? |
| --- | --- | --- | --- | --- | --- | --- | --- | --- | --- | --- | --- | --- |
| 1 | 3 | No | In service | No | No | Yes | Yes | 7 | 15 | 25 | No | No |
| 2 | 2 | No | In service | No | No | Yes | Yes | 6 | 15 | 45 | No | Yes |
| 3 | 5 | Yes | In service | Yes | No | Yes | Yes | 3 | 15 | 45 | No | Yes |
| 4 | 5 | No | In service | No | No | Yes | Yes | 11 | 12 | 45 | Yes | No |
| 5 | 3 | Yes | Out of service | Yes | No | Yes | Yes | 1 | 12 | 45 | No | Yes |
| 6 | 6 | Yes | Out of service | No | Yes | Yes | No | 7 | 15 | 45 | No | Yes |
| 7 | 6 | No | In service | No | No | No | No | 11 | 10 | 45 | Yes | No |
| 8 | 10 | Yes | In service | Yes | Yes | Yes | No | 3 | 10 | 45 | No | No |
| 9 | 6 | Yes | In service | Yes | Yes | Yes | No | 6 | 12 | 45 | No | Yes |
| 10 | 7 | Yes | In service | Yes | Yes | Yes | Yes | 8 | 15 | 45 | Yes | No |
| 11 | 8 | Yes | In service | No | Yes | Yes | Yes | NaN | 12 | 45 | Yes | No |
| 12 | 8 | Yes | In service | No | No | Yes | Yes | 1 | 12 | 45 | Yes | No |
| 13 | 8 | Yes | In service | Yes | Yes | Yes | Yes | 2 | 12 | 45 | Yes | No |
| 14 | 8 | Yes | In service | No | Yes | Yes | Yes | 4 | 24 | 45 | Yes | No |
| 15 | 8 | Yes | In service | Yes | No | Yes | Yes | 5 | 30 | 45 | Yes | No |
| 16 | 8 | Yes | In service | No | Yes | Yes | Yes | 6 | 36 | 45 | Yes | No |
| 17 | 8 | Yes | In service | No | No | Yes | Yes | 6 | 36 | 45 | Yes | No |
| 18 | 1 | No | In service | No | No | Yes | No | 9 | 15 | 45 | No | No |
| 19 | 5 | No | In service | No | No | Yes | Yes | 4 | 15 | 45 | No | Yes |
| 20 | 2 | No | In service | No | No | Yes | Yes | 3 | 15 | 45 | Yes | No |
| 21 | 5 | No | In service | No | No | Yes | Yes | 10 | 14 | 45 | No | Yes |
| 22 | 4 | No | In service | No | No | Yes | Yes | 4 | 15 | 25 | No | Yes |
| 23 | 2 | Yes | In service | Yes | No | Yes | Yes | 5 | 24 | 45 | Yes | No |
| 24 | 5 | Yes | In service | Yes | No | Yes | Yes | 4 | 12 | 45 | No | Yes |
| 25 | 1 | No | In service | No | No | Yes | No | 17 | 20 | 45 | No | Yes |
| 26 | 3 | No | In service | No | No | Yes | Yes | 10 | 15 | 45 | No | Yes |
| 27 | 4 | No | In service | No | No | Yes | No | 20 | 15 | 45 | Yes | No |
| 28 | 4 | Yes | In service | No | Yes | Yes | Yes | 7 | 10 | 45 | Yes | No |
| 29 | 14 | Yes | In service | Yes | No | Yes | Yes | 5 | 10 | 45 | Yes | No |
| 30 | 7 | Yes | In service | Yes | No | Yes | Yes | 5 | 10 | 45 | No | No |
| 31 | 14 | No | In service | No | No | Yes | Yes | 5 | 18 | 45 | Yes | No |
